# Evaluating pharmaceuticals and other organic contaminants in the Lac du Flambeau Chain of Lakes using risk-based screening techniques

**DOI:** 10.1371/journal.pone.0286571

**Published:** 2023-06-02

**Authors:** Matthew A. Pronschinske, Steven R. Corsi, Celeste Hockings

**Affiliations:** 1 Upper Midwest Water Science Center, U.S. Geological Survey, Madison, Wisconsin, United States of America; 2 Water Resource Program, Lac du Flambeau Band of Lake Superior Chippewa Indians, Lac du Flambeau, Wisconsin, United States of America; Tsinghua University, CHINA

## Abstract

In an investigation of pharmaceutical contamination in the Lac du Flambeau Chain of Lakes (hereafter referred to as “the Chain”), few contaminants were detected; only eight pharmaceuticals and one pesticide were identified among the 110 pharmaceuticals and other organic contaminants monitored in surface water samples. This study, conducted in cooperation with the Lac du Flambeau Tribe’s Water Resource Program, investigated these organic contaminants and potential biological effects in channels connecting lakes throughout the Chain, including the Moss Lake Outlet site, adjacent to the wastewater treatment plant lagoon. Of the 6 sites monitored and 24 samples analyzed, sample concentrations and contaminant detection frequencies were greatest at the Moss Lake Outlet site; however, the concentrations and detection frequencies of this study were comparable to other pharmaceutical investigations in basins with similar characteristics. Because established water-quality benchmarks do not exist for the pharmaceuticals detected in this study, alternative screening-level water-quality benchmarks, developed using two U.S. Environmental Protection Agency toxicological resources (ToxCast database and ECOTOX knowledgebase), were used to estimate potential biological effects associated with the observed contaminant concentrations. Two contaminants (caffeine and thiabendazole) exceeded the prioritization threshold according to ToxCast alternative benchmarks, and four contaminants (acetaminophen, atrazine, caffeine, and carbamazepine) exceeded the prioritization threshold according to ECOTOX alternative benchmarks. Atrazine, an herbicide, was the most frequently detected contaminant (79% of samples), and it exhibited the strongest potential for biological effects due to its high estimated potency. Insufficient toxicological information within ToxCast and ECOTOX for gabapentin and methocarbamol (which had the two greatest concentrations in this study) precluded alternative benchmark development. This data gap presents unknown potential environmental impacts. Future research examining the biological effects elicited by these two contaminants as well as the others detected in this study would further elucidate the ecological relevance of the water chemistry results generated though this investigation.

## Introduction

Pharmaceuticals are recognized as aquatic contaminants of emerging concern (CECs) because they have been detected in surface waters around the world and sometimes occur at concentrations which may negatively impact aquatic life [1, 2]. Pharmaceuticals are used daily around the world and are typically designed to have highly specific effects, such as antibiotics that target bacterial infections and anticonvulsants that are designed to treat seizures. Although pharmaceuticals have targeted effects, they often persist beyond their intended targets and are passed through the digestive systems of human or domestic animal targets into waste which retains the potential for eliciting biological activity if not effectively treated [3–5]. There are many potential sources of pharmaceuticals to the environment, such as effluent from pharmaceutical production facilities [6], improperly disposed of drugs [7], domestic animal waste [8], septic systems [9], and wastewater treatment plant (WWTP) effluent [4]. Pharmaceutical contamination is commonly associated with highly developed areas, high population densities, and/or watersheds with WWTP effluent contributions [10]. WWTPs are designed to capture and treat waste from many households; however, they are not required or designed to treat for all pharmaceuticals, and removal efficiencies vary for contaminants [4, 11–14]. As a result, land application of human biosolids, WWTP lagoons, and WWTP effluent outfalls are common sources of pharmaceuticals to the environment globally.

Although pharmaceuticals are designed to affect biological targets that are highly conserved across different taxa, their effects on nontarget organisms are not well-known. Available research indicates that pharmaceuticals possess the potential to adversely affect aquatic life [15]. In addition, other contaminants such as pesticides, surfactants, flame retardants, polycyclic aromatic hydrocarbons, plastics components, and corrosion inhibitors often co-occur with pharmaceutical compounds, resulting in a complex mixture of potentially harmful chemicals [16–19]. Despite these facts, few pharmaceuticals have established water-quality benchmarks. Because the potency of each contaminant is unique, concentration values alone do not impart biological relevance; highly potent contaminants may elicit adverse effects at low concentrations. Established water-quality benchmarks are typically used as a means of normalizing observed environmental concentrations by those known to elicit an adverse effect; however, in their absence alternative sources of toxicological information can be considered and used to estimate the potential for biological effects. Two such sources are the Toxicity Forecaster (ToxCast) program [20–23] and the ECOTOXicological Knowledgebase (ECOTOX) [24], both administered by the U.S. Environmental Protection Agency. The ToxCast program uses a standardized set of in vitro, high-throughput assays to evaluate the interactions or effects of a chemical on cells and subcellular components. The ECOTOX knowledgebase is an assemblage of primarily in vivo toxicity testing results from primary-literature references for thousands of chemicals. ECOTOX includes apical effect study results similar to those used to develop traditional aquatic-life benchmarks. Alternative benchmarks can be derived from data within these two sources and used to determine concentrations of concern for risk-based screening-level analysis of chemicals detected in environmental matrices [16].

This study was conducted in cooperation with the Lac du Flambeau Band of Lake Superior Chippewa Indians (hereafter referred to as “the Tribe”) whose tribal boundary spans across three counties (Vilas, Oneida, and Iron) in northern Wisconsin. Located in the southwest corner of Vilas County, the Lac du Flambeau Chain of Lakes is a critical component of the subsistence lifestyle for Tribe members who hunt, gather, and fish in the surrounding ecosystem. Additionally, the area attracts substantial summer tourism which is vital for the local economy, and vacation homes surround lakes along the Chain. Because the area is reliant on the Chain, water quality concerns are a high priority to the Tribe. One such concern is organic chemical contamination, including pharmaceutical compounds.

Although the Lac du Flambeau area is not highly developed currently, pharmaceuticals and other contaminants have been detected in other areas with similar land cover and wastewater characteristics [10, 25, 26]. Some of the more obvious potential sources of contamination in this area include leaks or failures in septic systems of the properties surrounding the Chain, chemical leachate from septic tanks or drain fields, possible leaks in the municipal wastewater conveyance system, discharge from the stormwater collection system, remnants of community wastewater treated by the Lac du Flambeau Water and Sewer Department, and runoff from lawn and agricultural areas. The Lac du Flambeau Water and Sewer Department previously utilized a facultative lagoon to treat the community’s wastewater, and effluent from the lagoon discharged into a small, unnamed wetland before flowing into Moss Lake. Further, a previously conducted U.S. Geological Survey (USGS) study indicated the potential for groundwater flow from the lagoon into Moss Lake [27]. This groundwater flow connection would leave the water quality of Moss Lake and other lakes in the Chain susceptible to influence from the WWTP lagoon. However, on October 28, 2020, the Lac du Flambeau WWTP system was substantially upgraded; further details regarding the upgrades are included in the Supplemental Information. These improvements may reasonably be expected to reduce the potential influence of the WWTP on the surrounding environment. Still, as a cautious measure, the Lac du Flambeau tribe sought to conduct a synoptic evaluation to assess the presence and effects of pharmaceuticals.

This contaminant monitoring study was conducted to meet the following objectives: 1) evaluate the prevalence of pharmaceuticals and other organic contaminants in the Lac du Flambeau Chain of Lakes (specifically, Fence Lake, Crawling Stone Lake, Moss Lake, Long Lake, Pokegama Lake, and Flambeau Lake), 2) evaluate the potential for detected contaminants to elicit adverse effects within the Chain’s aquatic life, 3) compare findings of the present study with similar studies to provide context to these results. By conducting a synoptic pharmaceutical sampling study and using alternative water-quality benchmarks, the detected contaminants were evaluated, and the most highly impacted sites were identified. The outcomes of this study are intended to inform the Tribe of the condition of contamination from pharmaceuticals and other organic contaminants within the Chain and to inform their water resources management decisions.

## Methods

### Sampling design and collection

**Fig 1:**
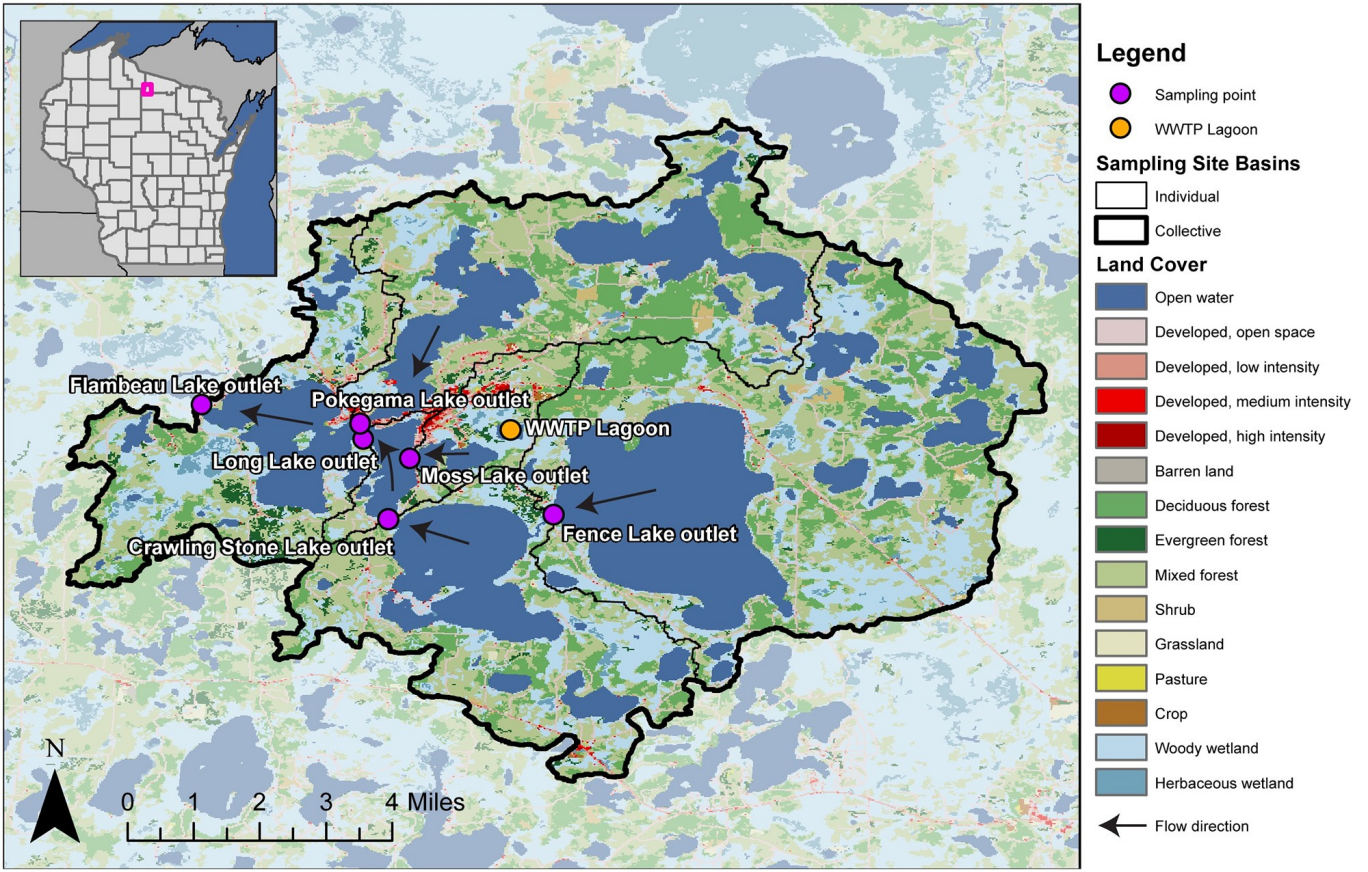
Sampled sites and basins. Sampled sites and basins within the Lac du Flambeau Chain of Lakes, Wisconsin, USA, August 2020 –May 2021. The Lac du Flambeau wastewater treatment plant (WWTP) lagoon and arrows indicating general flow direction between sampling sites are included as visual references. The map’s base layer was derived from the 2019 National Land Cover Database [28]. Drainage areas were delineated using USGS StreamStats [29] and the Watershed Boundary Dataset [30].

For this study, surface water samples were collected quarterly for one year (1: August 2020, 2: November 2020, 3: February 2021, and 4: May 2021) at six sites along the Lac du Flambeau Chain of Lakes (Fig 1). Study site locations were selected in channels between lakes to characterize the water flowing through the Chain before it empties into the Bear River at Flambeau Lake Outlet, the most downstream sampling site. A geographic information system was used to determine the drainage area and land cover of each of the sampling sites; further methodological details are included in the Supplemental Information. The drainage area of the sites ranged from 5.4 km^2^ (Moss Lake Outlet) to 146 km^2^ (Flambeau Lake Outlet), and the landcover of the drainage area was dominated by two broad classes: “Water and Wetland” which ranged from 37% to 55% and “Forest” which ranged from 40% to 46% (Table 1).

**Table 1:** Drainage area characteristics. Drainage area characteristics for six sites on the Lac du Flambeau Chain of Lakes, Wisconsin, USA sampled quarterly August 2020 –May 2021. USGS station numbers are recorded in the National Water Information System [31]. Drainage areas were delineated using USGS StreamStats [29] and the Watershed Boundary Dataset [30]. Land cover percentages were aggregated from the 2019 National Land Cover Database [28].

USGS Station Number	Station Name	Area (km^2^)	% Agriculture	% Forest	% Urban	% Water and Wetland	% Other
455659089515901	Fence Lake Outlet	56.8	0	40	4.4	54.6	1
455656089540501	Crawling stone Lake Outlet	83.1	0	40.4	4.7	53.9	0.9
455744089534801	Moss Lake Outlet	5.4	1.3	41.7	17.5	37.3	2.2
455801089542401	Long Lake Outlet	91.4	0.1	40	5.7	53.3	1
455811089542701	Pokegama Lake Outlet	32.5	0.1	46.2	6.8	44.9	2.1
455826089563101	Flambeau Lake Outlet	145.8	0.1	41.6	5.9	51.3	1.1

A quarterly sampling schedule was used in this study to capture potential seasonal variability which may be associated with increased population and recreation activity during the summer months or variability resulting from other environmental factors, such as precipitation, surface water runoff, water table levels, and temperature. Water samples were collected according to standard USGS methods [32] using Teflon and glass equipment that was properly cleaned prior to sample collection to prevent cross-contamination [33]; sample collection methods are further described in the Supplemental Information. One field replicate sample and one field blank sample were collected concurrently with the regular samples each quarter to evaluate potential bias and variability in sample collection or laboratory analysis processes.

### Laboratory analysis

Samples collected during this study were anlayzed for pharmaceuticals and related compounds at the USGS National Water Quality Labortory in Lakewood, Colorado, using the method of Furlong et al. [34], which is suitable for filtered water samples. The method includes 110 analytes, most of which are pharmaceuticals (S1 Table). In this method, a 100-microliter aliquot of the filtered water sample, previously amended with an aliquot of an isotope dilution standard (IDS) mixture of 20 stable-isotope labeled analyte analogues, is directly injected into a high-performance liquid chromatograph (HPLC) coupled to a triple quadrupole mass spectrometer (MS/MS) by using an electrospray ionization source operated in the positive ion mode. The analytes are separated on a 1.8-micron particle size, 30-mm x 100-mm C-18 reversed-phase HPLC column by using a reversed-phase gradient of formic acid/ammonium formate-modified water and methanol. The separated pharmaceuticals are ionized as the HPLC eluent stream enters the MS/MS interface as protonated molecular ions using positive electrospray ionization (positive ESI). Multiple reaction-monitoring (MRM) in the MS/MS produces two fragmentations of the protonated molecular ion of each pharmaceutical to produce two unique precursor-product ion pairs to identify each analyte specifically and sensitively. The primary MRM precursor-product ion transition is quantified for each pharmaceutical relative to the primary MRM precursor-product transition of a specific IDS selected, when possible, for its chemical similarity to the unlabeled analyte of interest. Recovery of water samples spiked with the suite of analytes determined by this method typically was greater than 90% in reagent, groundwater, drinking water, and surface water.

Method reporting limits (determined in reagent water) for individual pharmaceuticals ranged between 0.002 and 0.27 micrograms per liter (μg/L) during the period these samples were analyzed; the USGS National Water Quality Laboratory annually assesses reporting limits using a documented procedure [35, 36]. Further detail concerning reporting limits is included in the Supplemental Information. The median reporting limit for all analytes was 0.03 μg/L. The majority of detection limits for this method, as defined by the 25^th^ and 75^th^ percentiles of the method detection limit distribution, fall between 0.013 and 0.08 μg/L. Water chemistry laboratory analysis results were reviewed prior to data analysis. The concentrations of some chemicals were routinely reported as estimated values due to variability in method performance and other values were flagged as estimates according to USGS reporting procedure criteria [37] (Table 2, S2 Table). If matrix interference precluded the estimation of concentration (as determined by the USGS National Water Quality Laboratory), those values were removed from the analysis.

**Table 2:** Water chemistry results. Water chemistry results for chemicals detected in surface water samples collected from sampling sites on the Lac du Flambeau Chain of Lakes, Wisconsin, USA, August 2020-May 2021. Quarter 1: August 2020; Quarter 2: November 2020; Quarter 3: February 2021; Quarter 4: May 2021. In instances where the chemical was not quantifiable, that detection limit was used as an upper bound. Quantifiable concentrations are included in bold font. [μg/L = micrograms per liter, < = less than].

Station Name	Quarter	Acetaminophen (μg/L)	Atrazine (μg/L)	Caffeine (μg/L)	Carbamazepine (μg/L)	Fluconazole (μg/L)	Gabapentin (μg/L)	Metformin (μg/L)	Methocarbamol (μg/L)	Thiabendazole (μg/L)
Fence Lake Outlet	1	<0.01	**0.0232**	<0.043	<0.0022	<0.015	<0.08	<0.0066	<0.0056	<0.002
	2	<0.042	<0.01	<0.043	<0.0022	<0.015	<0.08	<0.0066	<0.0056	<0.002
	3	<0.042	**0.0209**	<0.043	<0.0022	<0.015	<0.08	<0.0066	<0.0056	<0.002
	4	<0.042	**0.0245**	**0.0185** ^1^	<0.0022	<0.015	<0.08	<0.0066	<0.0056	<0.002
Crawling Stone Lake Outlet	1	<0.01	**0.0231**	<0.043	<0.0022	<0.015	<0.08	<0.0066	<0.0056	<0.002
	2	<0.042	<0.01	<0.043	<0.0022	<0.015	<0.08	<0.0066	<0.0056	<0.002
	3	<0.042	**0.0181** ^1^	<0.043	<0.0022	<0.015	<0.08	<0.0066	<0.0056	<0.002
	4	<0.042	**0.0236**	<0.043	<0.0022	<0.015	<0.08	<0.0066	<0.0056	<0.002
Moss Lake Outlet	1	<0.01	**0.0116** ^1^	**0.0121** ^1^	<0.0022	<0.015	**0.308** ^1^	**0.0843**	<0.0056	<0.002
	2	<0.042	<0.01	<0.043	**0.00549** ^1^	<0.015	**0.326** ^1^	**0.0481**	<0.0056	<0.002
	3	<0.042	**0.00839** ^1^	<0.043	**0.00644** ^1^	<0.015	**0.282** ^1^	**0.046**	<0.0056	<0.002
	4	<0.042	**0.0111** ^1^	**0.0161** ^1^	**0.00511** ^1^	**0.0061** ^1^	**0.341** ^1^	**0.0293**	<0.0056	<0.002
Long Lake Outlet	1	<0.01	**0.0191** ^1^	<0.043	<0.0022	<0.015	<0.08	<0.0066	<0.0056	<0.002
	2	<0.042	**0.0167** ^1^	<0.043	<0.0022	<0.015	<0.08	<0.0066	<0.0056	<0.002
	3	<0.042	**0.022**	<0.043	<0.0022	<0.015	<0.08	<0.0066	<0.0056	<0.002
	4	<0.042	**0.02**	<0.043	<0.0022	<0.015	<0.08	**0.0118** ^1^	<0.0056	<0.002
Pokegama Lake Outlet	1	<0.01	**0.0118** ^1^	<0.043	<0.0022	<0.015	<0.08	<0.0066	<0.0056	<0.002
	2	<0.042	<0.01	<0.043	<0.0022	<0.015	<0.08	<0.0066	<0.0056	<0.002
	3	<0.042	**0.0107** ^1^	<0.043	<0.0022	<0.015	<0.08	<0.0066	<0.0056	<0.002
	4	**0.0805** ^1^	**0.0115** ^1^	**0.0201** ^1^	<0.0022	<0.015	<0.08	<0.0066	**0.142**	<0.002
Flambeau Lake Outlet	1	<0.01	**0.0163** ^1^	<0.043	<0.0022	<0.015	<0.08	<0.0066	<0.0056	<0.002
	2	<0.042	<0.01	<0.043	<0.0022	<0.015	<0.08	<0.0066	<0.0056	<0.002
	3	<0.042	**0.0156** ^1^	<0.043	<0.0022	<0.015	**0.0199** ^1^	<0.0066	<0.0056	**0.00242** ^1^
	4	<0.042	**0.0175** ^1^	<0.043	<0.0022	<0.015	<0.08	**0.0109** ^1^	<0.0056	<0.002

^1^ Laboratory remark codes of "estimated" were assigned to these values based on remarks from the USGS National Water Quality Laboratory.

No chemicals were detected in any of the field blank samples, indicating that sample collection and analysis methods did not introduce contamination (S3 Table). Among the four sets of replicate sample pairs collected in the field, there were 440 analyte comparisons (S4 Table). Of these comparisons, analytes were not detected in either sample for 429 instances, and 3 of the comparisons were precluded by interference during laboratory analysis. In two of the comparisons, an analyte was detected in one of the two replicate samples: metformin was detected at a concentration lower than all regular sample results in this study, and it occurred below the reporting level in this case. As a result, there is uncertainty associated with this low-level detection. Lidocaine was not detected in any samples collected for this study aside from the single replicate sample detection. Due to the low certainty associated with this detection, it was excluded from formal analysis. In these two cases, the detected concentration of metformin was 43% above its detection level, and lidocaine was 190% above its detection level, but both concentration values were flagged by the USGS National Water Quality Laboratory as estimates. There were six comparisons in which chemicals were detected in both samples; among these comparisons, the maximum relative percent difference was 17%, and the average was 8%. No adjustments were made to sample data as a result of replicate sample comparisons.

### Alternative benchmarks

Because contaminants have different inherent potencies, water-quality benchmarks are often used to put concentration data into context for assessment of potential biological effects. In the absence of established water-quality benchmarks, two U.S. Environmental Protection Agency databases (ToxCast and ECOTOX) were queried to gather relevant response and effect data for the detected contaminants. These data were collated by chemical and are hereafter referred to as “screening values.” Subsets of screening values were used to derive screening-level alternative water-quality benchmarks (hereafter referred to as “alternative benchmarks”) for seven of the detected contaminants according to previously published methods [16]. Summarized descriptions of screening value and alternative benchmark development methods are included below, and more information can be found in the previously published methods [16]. Using these alternative benchmarks, the biological relevance of observed pharmaceutical concentrations was estimated.

#### ToxCast

The ToxCast Program, facilitated by the U.S. Environmental Protection Agency Center for Computational Toxicity and Exposure, has characterized biological activities associated with more than 9,000 individual chemicals using several hundred high-throughput assays to monitor effects on cells, proteins, DNA, RNA, mitochondria, receptors, enzymes, etc. [38]. ToxCast in vitro high-throughput assays evaluate a wide-range of cellular and subcellular bioeffects; however, the assays do not necessarily cover all pathways. As a result, perturbations recognized in an apical study may not be recognized through ToxCast assays, and concentrations eliciting bioeffects recognizable at cellular and subcellular levels may not translate into apical effects typically used to define traditional water-quality benchmarks. Further, ToxCast assays were developed with an emphasis on human health and did not necessarily focus on aquatic species with dissimilar response pathways, especially plants and invertebrate organisms, and thus may miss perturbations that may affect those organisms.

Assays were gathered from version 3.2 of the ToxCast database [39], which was accessed using the ToxEval R-package [40], and processed following previously published methods [41, 42]. The assays used to develop these alternative benchmarks were chosen with consideration to data quality remarks, examination of dose-response curves, and the reliability of the assay to detect gain or loss of response signals; further details concerning this process are included in the Supplemental Information. Additionally, because some assays are not relevant for this study’s objectives, the nature of the assays and their redundancy were considered with a more holistic view of other information in ToxCast and the purposes of this study. The results of this curation process are included in S5 Table. The list of assays used in this study is included in S6 Table. Finally, the list of assays specifically excluded from this analysis is provided in S7 Table.

To develop alternative benchmarks from these screening values, a ToxCast summary metric was used: the activity concentration at cutoff (ACC) value, a metric which conveys the concentration at which an assay’s response first exceeds the baseline response value [38, 43]. Using ToxCast data, Exposure-Activity Ratio (EAR) values were calculated as quotients of observed concentrations and the ACC values from ToxCast assays (Eq 1). To assess all relevant assays for each chemical, EAR values were summed for each chemical (Eq 2). This does not necessarily mean that the effects measured in assays are additive, but the sum of EAR values can be useful for prioritizing among chemicals and samples to evaluate their potential to elicit biological effects. Because the number of relevant assays conducted varied between 131 and 446 for the detected chemicals (S5 Table), EAR_Chem_ values may be biased towards chemicals with greater amounts of relevant assays; however, this bias was found to be minimal after evaluation (S1 Fig). Through this process, seven of the nine detected chemicals were found to have assays suitable for usage as screening-level alternatives to water-quality benchmarks.

$$\text{EAR}=\frac{\text{Measured}\;\text{concentration}\;\text{in}\;\text{sample}\;(\mu \text{M})}{\text{ACC}\;\text{for}\;\text{chemical}-\text{assay}\;\text{pair}\;(\mu \text{M})}$$ (1)

$${\text{EAR}}_{\text{Chem}}=\sum {\text{EAR}}_{\left[i\right]}$$ (2)

where i = assays relevant for each individual chemical.

#### ECOTOX

The ECOTOX knowledgebase, also facilitated by the U.S. Environmental Protection Agency Center for Computational Toxicity and Exposure, was developed to serve as a cost-effective means of compiling toxicological data for benchmark development, chemical prioritization, and additional purposes [24]. ECOTOX is focused on in vivo toxicity assessments of apical effects and contains more than 50,000 references with over one million results for thousands of chemicals from which endpoint data have been systematically gathered and curated. In a similar previous study, endpoint concentrations from ECOTOX were assessed with regard to endpoint type, species diversity, and environmental persistence to derive alternative screening-level water-quality benchmarks for pharmaceuticals and related organic contaminants [16]. Although screening values were gathered for all nine of the contaminants detected in the present study, only seven included the information necessary for alternative benchmark development (S8 Table). Full ECOTOX benchmark derivation details are included in the previous study [16], and the methods are summarized within the Supplemental Information. These benchmarks were used to compute Toxicity Quotients (TQs) as the quotient of observed environmental concentrations and ECOTOX alternative benchmark values (Eq 3). Due to variability in the type and number of ECOTOX screening values available for each chemical, the minimum ECOTOX benchmark was used to calculate the maximum TQ value for each chemical detection (rather than summing TQ values as EAR values were in Eq 2).


$$TQ=\frac{\text{Measured}\;\text{concentration}\;\text{in}\;\text{sample}\;(\mu \text{g}/\text{L})}{\text{ECOTOX}-\text{derived}\;\text{benchmark}\;\text{for}\;\text{chemical}\;(\mu \text{g}/\text{L})}$$ (3)

### Hazard quotients

Hazard quotients (i.e., EARs and TQs) were computed by dividing each observed contaminant concentration by the alternative benchmarks of the respective contaminant (Eq 1; Eq 3). A hazard quotient value greater than one indicates that environmentally observed concentrations are greater than the alternative benchmark concentrations, suggesting that adverse biological effects may occur at these levels. However, given uncertainties associated with benchmark derivation and considering that the quarterly sampling approach used in this study was not likely to capture maximum contaminant concentrations, lower-level thresholds were set for these hazard quotients as a conservative approach to identify “priority” contaminants of potential concern (i.e., contaminants with the greatest potential for biological effects) and to rank them relative to one another. To accomplish this relative prioritization, utilizing both sets of alternative benchmarks is advantageous; however, because ToxCast and ECOTOX benchmarks are derived from information sources with substantial dissimilarities, direct comparisons are not necessarily appropriate. ECOTOX endpoints and the benchmark derivation process applied to these data are more comparable to apical effect studies and derivation processes used to define some traditional water-quality benchmarks. Therefore, the TQ prioritization threshold was set at 0.1 in order to maintain consistency with similar, previously conducted studies and other conservative risk assessments [16, 42]. Previous studies recognizing these dissimilarities evaluated comparisons between EARs and TQs and found that using an EAR prioritization threshold of 0.001 was comparable to a TQ prioritization threshold of 0.1 [16, 17, 42, 44, 45]. Therefore, detections at concentrations resulting in EAR values greater than 0.001 and/or TQ values greater than 0.1 were recognized as priorities. Hazard quotients and concentrations were compared amongst chemicals in some cases. Wilcoxon rank sum tests were conducted for these comparisons, and a threshold of p < 0.05 was used to determine statistical significance.

## Results

### Occurrence and magnitude

**Fig 2:**
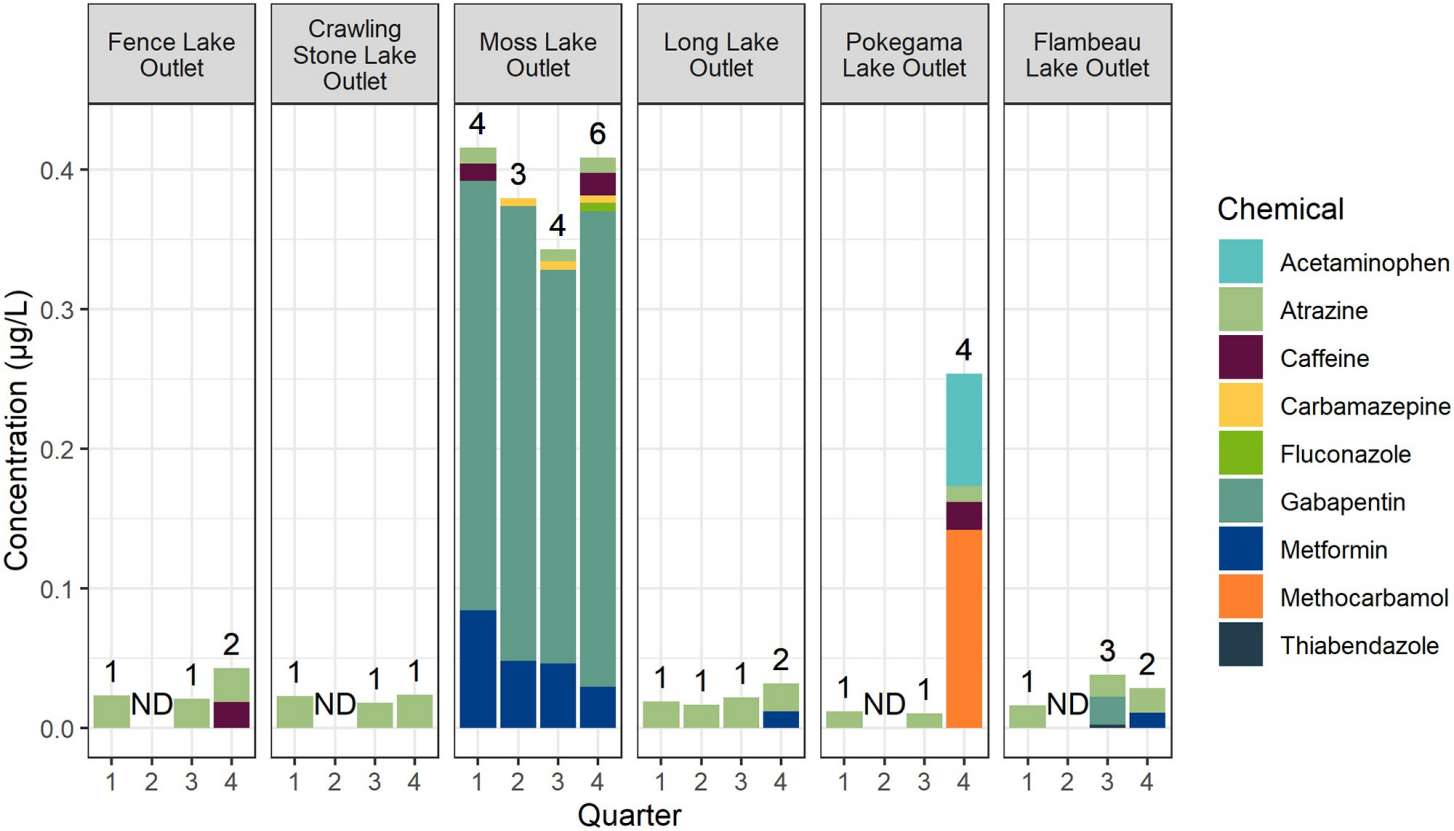
Total sample concentrations. Total concentrations from all quantified chemicals in samples collected at six sites on the Lac du Flambeau Chain of Lakes, Wisconsin, USA, August 2020 –May 2021. Quarter 1: August 2020; Quarter 2: November 2020; Quarter 3: February 2021; Quarter 4: May 2021. Bars have been grouped by site and colored by chemical composition. The number of unique chemicals detected in each sample is indicated above each bar. “ND” was used as a label for samples which lacked chemical detections.

In this study, only 9 contaminants (8 pharmaceuticals and 1 pesticide) were detected from among the 110 analytes monitored in 24 samples (S2 Table), and sample data can be accessed using the Nation Water Information System [31]. The number of contaminants detected at each site ranged from 1 to 6 (Crawling Stone Lake Outlet, 1; Fence Lake Outlet and Long Lake Outlet, 2; Pokegama Lake Outlet and Flambeau Lake Outlet, 4; Moss Lake Outlet, 6). The quarterly samples collected in May 2021 had the greatest number of detections (17) whereas the fewest chemicals (4) were detected in November 2020 quarterly samples. Four contaminants were detected only once: acetaminophen, fluconazole, methocarbamol, and thiabendazole. Five additional contaminants were detected on more than one occasion: atrazine (19 detections), metformin (6 detections), gabapentin (5 detections), caffeine (4 detections), and carbamazepine (3 detections). Concentrations for detected contaminants ranged from 0.002 μg/L (thiabendazole) to 0.34 μg/L (gabapentin). Atrazine, a pesticide, was detected in every sample, except for those from the second quarter (November 2020), during which atrazine was only detected in the sample collected from Long Lake Outlet, suggesting seasonality in the occurrence of atrazine. The detected concentrations of atrazine were relatively consistent across all samples, ranging from 0.008 μg/L to 0.025 μg/L (Fig 2, S1 Table). If analytical results from only pharmaceutical compounds are considered (by excluding atrazine), 15 of the 24 samples (62.5%) included no detections. Detection data are summarized in Fig 2 and Table 2. Additionally, lidocaine was detected in a single replicate sample, but it was not included in formal evaluations and results due to the low certainty associated with this occurrence (S4 Table).

Moss Lake Outlet was the site with the greatest contaminant detection frequency and the greatest sample concentrations. Gabapentin and metformin were detected in every sample at Moss Lake Outlet, and they occurred at greater concentrations than those observed in all other samples in the study. Atrazine and carbamazepine were each detected in three of the four samples from Moss Lake Outlet, and caffeine was detected in half of the Moss Lake Outlet samples. The sole detection of fluconazole within this study occurred at Moss Lake Outlet. The fourth quarter (May 2021) sample at Pokegama Lake Outlet stood out due to the sole detections of methocarbamol and acetaminophen (Fig 2, Table 2).

### Potential biological effects

**Fig 3:**
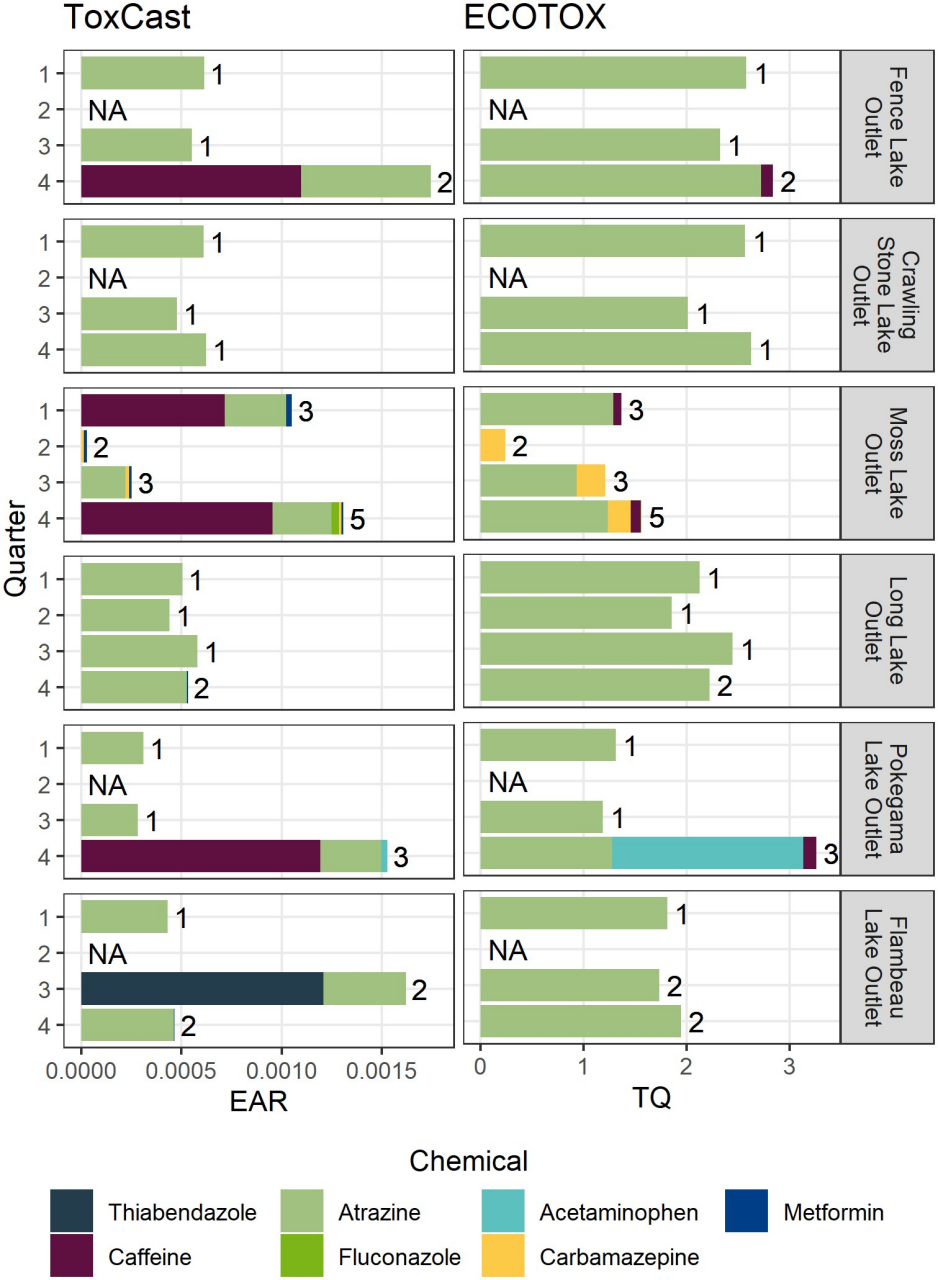
Total hazard quotient values. Total hazard quotient values for contaminants detected in samples collected quarterly in the Lac du Flambeau Chain of Lakes, Wisconsin, USA, August 2020 –May 2021. ToxCast assay exposure-activity ratios (EARs, left) were considered “priorities” if they exceeded the threshold of 0.001. ECOTOX derived benchmark toxicity quotients (TQs, right) were considered “priorities” if they exceeded the threshold of 0.1. Bars have been grouped by site and colored by chemical composition. The number of unique chemicals represented by the respective hazard quotient in each sample is indicated to the right of each bar. “NA” was used as a label for samples which lacked chemical detections or alternative benchmarks from which to compute hazard quotients.

Seven of the nine contaminants detected in this study were represented by screening-level alternative benchmarks derived from ToxCast and ECOTOX (Fig 3). Methocarbamol and gabapentin were not represented by either source of alternative benchmarks. The EAR_Chem_ values (computed using ToxCast alternative benchmarks) ranged nearly three orders of magnitude, from 3.52 x 10^−6^ to 1.21 x 10^−3^ (Fig 3). Caffeine and thiabendazole occurred at levels above the EAR_Chem_ threshold. TQ values (computed from ECOTOX alternative benchmarks) ranged nearly five orders of magnitude, from 3.41 x 10^−5^ to 2.72 (Fig 3). Atrazine, acetaminophen, and carbamazepine were detected at concentrations exceeding the TQ threshold. Although atrazine concentrations were not significantly greater than the rest of the detected contaminants, atrazine TQ values were significantly greater than all other TQ values in the study. EAR threshold exceedances occurred at three sites (Fence Lake Outlet, Flambeau Lake Outlet, and Pokegama Lake Outlet). TQ threshold exceedances occurred at all six sites (Fig 3).

## Discussion

### Occurrence and magnitude

In general, the detection frequencies and concentration levels for the sites and contaminants monitored in this study were low. The influence of the WWTP lagoon was not quantified in this study, but Moss Lake Outlet stands out as having substantially greater contaminant detection frequencies and concentrations than other sites. The elevated concentrations at Moss Lake Outlet indicate that contaminants could be leaching from the municipal wastewater treated by the facultative lagoon into this lake. However, even the elevated Moss Lake Outlet sample concentrations are comparable to sample results from two similar studies which are described in further detail below [16, 25]. The relatively low degree of influence exhibited by the WWTP lagoon adjacent to this Chain may be due in part to dilution and potential infiltration from the wetland which the lagoon discharges into before entering the lake [46]. Further, the pharmaceutical concentrations observed in this study may be the result of residual contamination from wastewater treated before the system was upgraded in October 2020. The upgraded treatment system and processes (further described in the Supplemental Information) may result in even fewer pharmaceutical detections and/or lower concentrations than those observed in this study. Additionally, individuals can limit their contributions to contamination through mindful usage and disposal of chemicals. For example, pharmaceutical contamination could be reduced through proper disposal of old and unused pharmaceuticals, and pesticide contamination could be reduced by minimizing chemical applications and timing their applications appropriately [7, 47].

The same suite of contaminants that were monitored in this study were also monitored in a recent investigation of 44 tributaries in the nearby Great Lakes basin [16]. The number of detected contaminants and the maximum sample concentrations at each site on the Chain (including Moss Lake Outlet) were in the bottom 20^th^ percentile of maximum sample concentrations among the more diverse Great Lakes tributary sites [16]. It should be noted that differences in lake and stream hydrology obfuscate some comparisons, and most sites within the Great Lakes tributary study included greater degrees of developed land cover and might reasonably be expected to have a greater degree of pharmaceuticals present [10]. However, some sites, including those on the Menomonee River, St. Louis River, and Bad River had similar watershed characteristics and yielded similar sample results, suggesting that the contaminants and concentrations detected in this study are not inordinate (S2 Fig).

Another pharmaceutical contamination study, which was recently conducted in Minnesota and included undeveloped lakes, also yielded similar overall results: low-level detections of several wastewater-related contaminants despite low degrees of potential wastewater influence. Eight of the contaminants monitored in the present study (acetaminophen, caffeine, carbamazepine, metformin, thiabendazole, amphetamine, hydrocodone, and venlafaxine) were also monitored through passive water samplers, sediment samples, and fish tissue samples in the Minnesota Lakes [25]. Caffeine and metformin, which were detected in surface water samples from Lac du Flambeau, were also detected in fish tissue samples from the undeveloped lakes of the Minnesota study [25]. Acetaminophen, carbamazepine, and thiabendazole were detected in Lac du Flambeau samples but not any of the samples from the undeveloped lakes of the Minnesota study. Conversely, amphetamine, hydrocodone, and venlafaxine were detected in passive water samplers deployed in undeveloped lakes in the Minnesota study, but they were not detected in the surface water samples from Lac du Flambeau [25]. Variability in the specific contaminants detected in these two studies could be influenced by many factors, including different respective sources of these contaminants, hydrologic connectivity of sources, the media monitored, monitoring methods, and/or laboratory methods. Despite these differences, both studies presented low-level detections of few contaminants.

Other interesting aspects of the chemical detection frequencies and concentrations observed in this study included the pronounced detections at the Pokegama Lake Outlet in May 2021 and the detection patterns observed for atrazine. Aside from the elevated concentrations at Moss Lake Outlet, the fourth quarter sample (May 2021) collected from Pokegama Lake Outlet had the highest total sample concentration, which suggests a potential seasonal influence or periodic influence given the sole detections of methocarbamol (0.142 μg/L) and acetaminophen (0.0805 μg/L). These two concentrations were among the greatest detected concentrations in the study. The Pokegama Lake watershed lacks WWTP effluent contributions, but it does include septic systems for private properties adjacent to the lake, some of which are vacation homes that are more commonly occupied during warmer months. It was also interesting that despite the Chain’s drainage area containing little agricultural land (<1%; Table 1), the herbicide atrazine was frequently detected and occurred in 79% of samples. The source of atrazine in these samples may be a combination of runoff from agricultural areas, chemical application on residential and vacation properties, and atmospheric deposition. Atrazine has been recognized as one of the most frequently detected pesticides in rainfall, and it has been found to be transported from agricultural areas to non-agricultural areas [48, 49]. Also, similar to other studies, atrazine exhibited a seasonal pattern across the sites in this study; quarter two (November 2020) had the fewest detections and lowest concentrations [50].

### Potential biological effects

Caffeine and thiabendazole were detected at concentrations that exceeded the EAR threshold (Fig 3). These threshold exceedances were primarily attributable to EAR values from assays designed to detect alterations to DNA binding to help understand changes in reporter genes as related to the genes SOX1 and Cyp1A1 (ATG_Sox_CIS_up and CLD_CYP1A1_24hr assays, respectively). More detailed descriptions of these assays and endpoints can be found by searching for them within the CompTox dashboard [51]. Caffeine also exceeded the TQ threshold based upon a study which observed elevated superoxide dismutase activity in the liver of goldfish (*Carassius auratus*) at concentrations of 3.2 μg/L; this elevated activity indicates that severe oxidative damage occurred within the liver [52]. The detection of acetaminophen also resulted in a TQ threshold exceedance, and that alternative benchmark was derived from a study reporting histopathological damage in the kidneys and livers of zebrafish (*Danio rerio*), increased embryo abnormalities (spinal deformation and pericardial edema), and increased embryo mortality at a time-weighted average concentration of 0.87 μg/L [53]. TQ values for carbamazepine detections also exceeded the prioritization threshold in some cases and were calculated using a benchmark derived from a study that reported significant mRNA changes of enzymes and proteins involved in the prevention of oxidative stress, biotransformation, and reversible protein posttranslational modification at an exposure concentration of 0.23 μg/L [54].

Despite the primary focus of pharmaceutical compounds in this study, atrazine emerged as the contaminant with the greatest potential for biological effects. Though the detected concentration of atrazine did not exceed the EAR threshold, the top 11 greatest TQ values are all attributable to atrazine. The endpoint used to derive the ECOTOX benchmark and TQ values for atrazine reported a significant reduction in the proportion of tadpoles (*Rana pipiens*) that reached metamorphosis at concentrations of 0.1 μg/L [55]. Additionally, 15 other endpoints from 7 other studies within ECOTOX reported that atrazine elicited effects on the mortality, growth, development, enzymes, injury, and physiology of various organisms including amphibians, algae, and fish at nearly identical concentrations (0.1–0.14 μg/L) (S8 Table). These endpoints and studies serve as multiple lines of evidence indicating a potential threat from atrazine to aquatic life in the Lac du Flambeau Chain of Lakes. Atrazine’s high toxic potential is not surprising because the chemical is known to be ubiquitous and has been cited as a contaminant of concern in previous studies [42, 44, 45, 49, 56].

Atrazine, caffeine, and thiabendazole are examples of highly potent contaminants detected in this study. Relative to other contaminants, atrazine was detected at moderate concentrations; however, atrazine exceeded the TQ threshold by more than an order of magnitude. Caffeine and thiabendazole serve as similar but less extreme examples of potent contaminants; they were detected at relatively low concentrations, but given their potency, as measured in ToxCast assays, they still exceeded EAR thresholds. Likewise, acetaminophen is a potent chemical; its concentration was moderate relative to others in this study, but its TQ value far exceeded the prioritization threshold. Conversely, metformin is an example of a low-potency contaminant. Its concentrations were among the greatest detected in this study, but it did not exceed TQ or EAR thresholds due to relatively high ToxCast and ECOTOX alternative benchmark values.

### Limitations

Although ToxCast and ECOTOX serve to fill gaps in toxicological information for chemicals without water-quality benchmarks, they are not comprehensive. Contaminant potency cannot be determined without an established or alternative water-quality benchmark, leaving gabapentin and methocarbamol without estimates of potential biological effects. Although there was not enough information to derive an alternative benchmark for methocarbamol, it should be noted that a single study was available in ECOTOX, and endpoint concentrations suggested that methocarbamol can be a highly potent contaminant. Schoenfuss et al. [57] observed adverse effects, including altered hepatosomatic and aggression indices and increased hepatocyte vacuolization in fathead minnows (*Pimephales promelas*) after aquatic exposure to 0.023 μg/L of methocarbamol. When comparing to the observed concentration of methocarbamol in the current study, this endpoint would have resulted in TQ values of 123, greater than the currently reported maximum TQ of 2.72 from atrazine. Similarly, though no relevant ECOTOX data were available for gabapentin at the time of this study, studies outside of ECOTOX reported effects at environmentally relevant concentrations. Effects, including increased activity of catalase (indicative of oxidative stress) and inhibition of genes whose expression regulate growth, development, and glucose metabolism, were observed in zebrafish (*Danio rerio*) at a concentration of 0.1 μg/L [58, 59]—lower than four of the five detected concentrations of gabapentin in this study, indicating the potential for adverse biological effects in the Lac du Flambeau Chain. Additionally, gabapentin-lactam, a transformation product of gabapentin, has been found to have greater toxicity and environmental persistence than its parent compound [60, 61]. Gabapentin-lactam was found to elicit adverse effects at concentrations as low as 0.01 μg/L [61], and it was observed in another study at a surface water concentration of 0.7 μg/L [60], suggesting that adverse effects are elicited at environmentally relevant concentrations. Although gabapentin-lactam was not monitored in this study, the presence of gabapentin indicates that this transformation product could pose a potential risk. Contaminants lacking the information necessary to derive alternative water-quality benchmarks, such as gabapentin and methocarbamol, maintain the potential to elicit adverse effects.

Additionally, it is possible that sensitive species and pathways were not captured by the assays within ToxCast and endpoints within ECOTOX. This is especially true for chemicals with few reference studies in ECOTOX. For example, though the hazard quotient values for metformin were quite low (two and three orders of magnitude less than the prioritization threshold), usage of the drug in humans has been tied to birth defects [62]. Given the presence of this contaminant in all sampling events at the Moss Lake Outlet site, additional information regarding the effects of metformin on aquatic organisms and even semi-aquatic organisms (amphibians and aquatic mammals) could be relevant with long-term chronic exposure to metformin.

Laboratory limits of chemical concentration quantification impose limitations on this study. Chemicals occurring near their detection levels may have greater uncertainty, as exemplified by the low-level detections of metformin and lidocaine in only one sample of a replicate sample pair (S4 Table). Contaminants with low alternative water-quality benchmarks may have occurred at concentrations too low to be quantified by these laboratory methods and yet have exceeded alternative benchmark concentrations. In other words, contaminants which were monitored but unquantifiable maintain the potential to elicit adverse effects. The detection limits for acetaminophen and atrazine were greater than their alternative benchmarks thresholds from ECOTOX, and the detection limit for caffeine was greater than both its ToxCast and ECOTOX alternative benchmark thresholds. As a result, any water concentration greater than or equal to the detection limits for these contaminants would have exceeded at least one alternative benchmark threshold. However, it should be noted that the water chemistry data provided by the USGS National Water Quality Laboratory [31] for this study included estimated concentrations that were below the detection limits for some contaminants; therefore, the possibility of quantifying these contaminants below their EAR and/or TQ thresholds remained. The detection limit for atrazine was an order of magnitude greater than its TQ threshold. Despite the contaminant occurring near (and, in one instance, below) its detection limit, each quantification of atrazine exceeded the TQ threshold considerably. The TQ threshold was exceeded by atrazine at all sites during quarters one (August 2020), three (February 2021), and four (May 2021). During quarter two (November 2020), atrazine concentrations may have dropped below quantification limits but may have remained at potentially harmful levels (above the TQ threshold) due to its high potency. Each detection of caffeine occurred below the method detection limit, so concentrations were considered estimates; however, three out of four occurrences exceeded ToxCast and/or ECOTOX benchmarks. Finally, another limitation is presented by the fact that few samples were collected from each site throughout the year, and due to hydrologic variability and other factors, sampled concentrations were likely not the maximum concentrations that occurred in each quarter. Had the maximum concentration of each contaminant been observed, other contaminants may have been detected and additional contaminants may have exceeded EAR and/or TQ thresholds.

## Summary and conclusions

Pharmaceuticals were not prevalent at most sites in this study, with more than 60% of samples containing no contaminants other than atrazine (a pesticide included within the analytical schedule). Only 9 of the 110 contaminants monitored in this study were detected: 8 pharmaceuticals (acetaminophen, caffeine, carbamazepine, fluconazole, gabapentin, metformin, methocarbamol, thiabendazole) and 1 pesticide (atrazine). Contaminant detections seemed to exhibit a seasonal pattern with peak concentrations in May 2021 and minimum concentrations during November 2020. The water chemistry results from this study had comparable concentration magnitudes and detection frequencies to those of similar studies of analogous watersheds. In the current study, pharmaceuticals were most frequently detected and had the greatest concentrations in samples from Moss Lake Outlet, suggesting that this site may be influenced by the adjacent WWTP lagoon which contains municipal wastewater from the Lac du Flambeau community. Due to a lack of established benchmarks for the monitored contaminants, alternative screening-level water-quality benchmarks were employed for estimating bioeffect potential from observed contaminant concentrations. Atrazine had the greatest likelihood of eliciting adverse biological effects due to its ubiquity in these samples and its high potency according to the alternative ECOTOX benchmark employed this study. Acetaminophen, caffeine, carbamazepine, and thiabendazole also occurred at concentrations that exceeded alternative benchmark hazard quotient thresholds. Further, because alternative benchmarks could not be developed for gabapentin and methocarbamol (which occurred at the greatest concentrations in this study), the full potential for biological effects is uncertain. Future studies investigating and confirming the toxicological potentials of the contaminants detected in this study would increase certainty in these estimates. Additionally, a study of pesticide contamination could provide critical insight into the potential impacts that hundreds of other chemicals may elicit, given the relative importance of atrazine in the current study. Finally, a follow-up study and continued monitoring of pharmaceuticals could elucidate the potential benefits of the wastewater treatment system upgrades implemented in this area.

## Supporting information

S1 Text Additional details on site background and study methods. Further details related to the wastewater treatment plant upgrade, sample collection methods, GIS methods, reporting limits, EAR derivation, and ECOTOX benchmark derivation.
(DOCX)

S1 Fig Comparison of maximum EAR to sum of EARs. Comparison of the maximum Exposure-Activity Ratio (EAR) values (computed from minimum Activity Concentration at Cutoff values) and the sum of EARs (computed from all relevant Activity Concentration at Cutoff values) for chemicals detected in samples from the Lac du Flambeau Chain of Lakes, Wisconsin, USA, August 2020 –May 2021. The shade of each point is part of a gradient representing the number of assays considered for computing the EARs of each chemical. For the purposes of this comparison, EARs were computed using a constant, nominal concentration of 1 μg/L for all chemicals.
(TIF)

S2 Fig Comparison of Lac du Flambeau sample concentrations to similar Great Lakes tributary sample concentrations. Surface water sample concentrations collected from the Lac du Flambeau Chain of Lakes, Wisconsin, USA, August 2020 –May 2021 (left). Surface water sample concentrations collected from three Great Lakes Tributary sites (USGS station IDs from top to bottom: 04024000, 04027000, 04087014), November 2017 –May 2018 (right). Sample concentration data are accessible from the National Water Information System. The three selected Great Lakes tributary sites capture drainage areas with similar wastewater contributions and land cover profiles. Gabapentin concentration values are all considered to be estimates. All samples were analyzed for using the same analytical procedures at the USGS National Water Quality Laboratory. The number of unique chemicals detected in each sample is indicated to the right of each bar. “ND” was used as a label for samples which lacked chemical detections.
(TIF)

S1 Table Chemical information. Chemicals and use classes monitored in the Lac du Flambeau Chain of Lakes, Wisconsin, USA, August 2020-May 2021.
(XLSX)

S2 Table Water chemistry results. Water chemistry data from samples collected from sampling sites on the Lac du Flambeau Chain of Lakes, Wisconsin, USA, August 2020-May 2021.
(XLSX)

S3 Table Blank sample results. Field blank sample results for samples collected in Lac du Flambeau Chain of Lakes, Wisconsin, USA, August 2020-May 2021.
(XLSX)

S4 Table Replicate sample results. Replicate sample results for samples collected in Lac du Flambeau Chain of Lakes, Wisconsin, USA, August 2020-May 2021.
(XLSX)

S5 Table ToxCast assay information. ToxCast assay information for chemicals detected in the Lac du Flambeau Chain of lakes, Wisconsin, USA, August 2020-May 2021.
(XLSX)

S6 Table ToxCast assays used. ToxCast assays used in computation of exposure-activity ratios from chemical analysis of surface water samples for pharmaceuticals in the Lac du Flambeau Chain of Lakes, Wisconsin, USA, August 2020-May 2021.
(XLSX)

S7 Table ToxCast assays excluded. List of ToxCast assay endpoint and chemical combinations that were excluded from the computation of exposure-activity ratios.
(XLSX)

S8 Table ECOTOX benchmarks. ECOTOX-derived water quality benchmarks developed for evaluation of chemicals detected in water samples collected from the Lac du Flambeau Chain of Lakes, Wisconsin, USA, August 2020-May 2021.
(XLSX)

S9 Table ECOTOX data. Data gathered from the aquatic ECOTOX knowledgebase for chemicals detected in the Lac du Flambeau Chain of Lakes, Wisconsin, USA, August 2020-May 2021.
(XLSX)

S10 Table ECOTOX data revisions. List of ECOTOX results that were manually adjusted or removed.
(XLSX)
