# Supplementary material for: Evaluating pharmaceuticals and other organic contaminants in the Lac du Flambeau Chain of Lakes using risk-based screening techniques
Source: PLoS One. 2023 Jun 2;18(6):e0286571. doi: 10.1371/journal.pone.0286571 (PMC10237466; doi:10.1371/journal.pone.0286571)
Supplement: S1 Text — Further details related to the wastewater treatment plant upgrade, sample collection methods, GIS methods, reporting limits, EAR derivation, and ECOTOX benchmark derivation. (DOCX) [file pone.0286571.s001.docx]

Evaluating pharmaceuticals and other organic contaminants in the Lac du Flambeau Chain of Lakes using risk-based screening techniques: supplemental information

Short title: Pharmaceuticals and other contaminants in the Lac du Flambeau Chain of Lakes

Matthew A. Pronschinske^1^*, Steven R. Corsi^1^, Celeste Hockings^2^

^1^Upper Midwest Water Science Center, U.S. Geological Survey, Madison, Wisconsin, USA

^2^Water Resource Program, Lac du Flambeau Band of Lake Superior Chippewa Indians, Lac du Flambeau, Wisconsin, USA

* Correspondence: mpronschinske@usgs.gov

# Wastewater treatment plant upgrade

On October 28, 2020, the Lac du Flambeau lagoon system was substantially upgraded to include primary and secondary facultative lagoon cells, a moving bed biofilm reactor system for nitrogen reduction, a settling basin for solids removal, and disinfection via ultraviolet light. After disinfection, the treated water is discharged to the subsurface by new seepage cells (which are further from lakes and have greater vertical separation from the water table than the old cells). The new cells enable additional treatment to take place in the soil before the water incorporates into the surrounding aquifer and surface water features. Further, whereas the old system required a seasonal surface water discharge, all water treated with the new system is discharged to the subsurface. Additionally, the clay liner in the secondary lagoon cell was replaced with an impermeable high-density polyethylene membrane liner to prevent partially treated water from entering the aquifer via leakage. These improvements may reasonably be expected to reduce the influence of the wastewater treatment plant on the surrounding environment.

# Sample collection methods

Equal-width increment (EWI) sampling was employed at each sampling site to collect a composite water sample from a lateral transect of the channel [1]. When discharge was too low for the collection of a true EWI sample, the EWI method was still adhered to in order to collect a composite sample that was laterally and vertically integrated across the channel transect. To prevent the contamination of samples from sampling equipment, Teflon equipment (sample bottle, sample nozzle) was used, and a 10 L glass jar was used to composite and homogenize the subsamples. Equipment was cleaned with phosphate-free soap (Liquinox) and tap water, sprayed with methanol, rinsed twice with deionized water, and finally rinsed with pesticide-grade blank water (MilliporeSigma OmniSolv, WX0004-1) according to methods described in the USGS National Field Manual [2]. Prior to collection, all sampling equipment was field-rinsed three times with water from the sampling site. Subsamples were collected from points along the lateral channel transect and emptied into the glass composite jar according to methods described in the USGS National Field Manual [1]. Next, the sample was processed by thoroughly homogenizing the composited sample and drawing a final subsample from the glass jar into a 20 mL plastic syringe. A 0.7 µm glass fiber filter (Whatman GMF, 6825-2517) was attached to the syringe and pre-conditioned by discharging 10 mL of sampled water before discharging the remaining 10 mL sample into a 20 mL, baked, amber-glass vial according to methods described in the USGS National Field Manual [3]. The samples were stored in the absence of light between 0 and 4 degrees Celsius and arrived at the laboratory for analysis within 48 hours of their collection.

Field replicate and field blank samples were collected to ensure the quality of water concentration data. For replicate samples, after the regular sample was drawn from the glass composite jar, the jar was emptied. A second set of subsamples was collected from the channel and emptied into the glass composite jar. Replicate samples were processed and analyzed using methods identical to regular samples. Blank samples were collected by passing pesticide-grade blank water (instead of sampled surface water) through clean sampling equipment (sample bottle, sample nozzle, and glass composite jar). The blank sample was processed and analyzed using methods identical to regular samples. The methods for collecting replicate and blank samples are further described in the USGS National Field Manual [1].

# GIS methods

After sampling points had been defined, StreamStats [4] was used to download shapefiles of the drainage area for each of the sites to load into a geographic information system. The extent of the drainage area was compared with NHDPlus linework [5] and the Watershed Boundary Dataset [6] for congruence. The StreamStats basins for Flambeau outlet and Pokegama outlet included an area that extended across a Watershed Boundary Dataset border. A USGS topographical map of that area was consulted, and the Watershed Boundary Dataset border was supported. The 2019 National Landcover Dataset was used to gather landcover percentages for each of the watersheds for the sites and aggregated to several Level I categories [7].

# Concentrations with respect to reporting limits

Atrazine, gabapentin, metformin, and methocarbamol were the only chemicals that were detected above their respective reporting levels. Caffeine, fluconazole, carbamazepine, and thiabendazole had mean detected concentrations that were lower than their respective RLs. While there is confidence that these chemicals were present, their concentrations have a greater degree of uncertainty than those with concentrations above the reporting level; therefore, the concentrations for these chemicals are considered to be estimates [8].

# EAR derivation and screening

ToxEval [9] was used to gather assay results from version 3.2 of the ToxCast Database [10] and evaluate the potential biological activities associated with each chemical detected in water samples collected for this study. Data analysis routines to define exposure-activity ratio (EAR) values for detected chemicals were similar to previously published methods [11,12]. ToxEval was used to filter ToxCast data by removing flagged data and cleaning endpoint information. Additionally, dose-response curves were examined; assays that seemed unreliable or responses that seemed to be of questionable quality were removed. Excluded assays or chemical-assay pairs are listed in S7 Table.

Extrapolating EAR information for application to environmental concentrations can be complex. For example, current information does not include correction for chemical partitioning in the assay system (e.g., free versus bound chemical in a test well), so actual biological activity in vitro or in situ may differ from this. However, it does provide a value that effectively normalizes for relative concentration detected in the environment and relative potency to elicit a specific biological effect. Thus, the EAR value is suitable for relative ranking and prioritization. Still, it is recognized that not all ToxCast assays used in this analysis are likely to be relevant to ecological species even though many of them target biological activities that are conserved among species. A complete evaluation of ToxCast assays for ecological relevance is currently not available, so the EAR approach used here is conservative in this respect.

# ECOTOX benchmark derivation

The EPA resource, ECOTOX knowledgebase [13], was used to develop screening-level alternative water-quality benchmarks in a previous study [14], and the methods are summarized here. ECOTOX is composed of a great diversity of test species, effects, and endpoints; however, the amount and types of endpoints gathered from ECOTOX varied widely among chemicals (S9 Table). Relevant endpoints from among these screening values were refined through a benchmark derivation process, similar to that of Hull et al. [15] in which application factors (AFs) were used to account for uncertainties associated with the screening values for each chemical. The overall AF was computed as the product of three discrete AF values: AF_Endpoint_, AF_Species_, and AF_Persistence_. The following data were required for the development of a comprehensive application factor: endpoint code, chemical persistence, and the variety of species tested in the toxicity studies gathered from the ECOTOX database.

The ECOTOX results were assigned to one of the three benchmark types (No Effect, Low Effect, and Acute Effect) depending upon the ECOTOX-reported endpoint code. The minimum concentration from a NOEC/NOEL or EC/LC ≤ 10 endpoint was generally used to define the No Effect benchmark type for each chemical. Similarly, the minimum concentration from a LOEC/LOEL or EC/LC 10 < x < 50 endpoint was generally used to define the Low Effect benchmark type for each chemical. Finally, the minimum concentration from an EC/LC50 endpoint was generally used to define the Acute Effect benchmark type for each chemical. No Effect and Low Effect benchmark types earned an ${AF}_{endpoint}$ of 10 [15]. Unlike Hull et al. [15], Acute Effect benchmark types earned an elevated ${AF}_{endpoint}$ of 100 due to the assumption that lower-level effects may be elicited at concentrations considerably lower than those which provoke acute effects.

Subsequently, an application factor for test-species diversity was applied to all three benchmark types for a chemical contingent upon whether the test-species diversity requirement was met. A chemical was considered to have met the species requirement if test-species reported in ECOTOX included, at a minimum, the following: three unique species of fish, three unique species of invertebrates, and one plant species. Using the test species information from ECOTOX, all test species were gathered into one of the three aforementioned groups. Although most test species fit into these categories well, those that did not were assigned to the species groups thought to be most suitable. Amphibian species were included within the “fish” species category, and species of algae were included within the “plant” species category for our purposes. Because a chemical that has been tested upon a variety of species is presumably better characterized than one not meeting the species requirement, compounds meeting the test species requirement earned an ${AF}_{species}$ of 1. However, if test species requirements were not met for a compound, an ${AF}_{species}$ of 2 was assigned, similar to Hull et al. [15].

Finally, chemical persistence in an aquatic environment was the last aspect considered in the assignment of AFs in the benchmark development process. Persistence AFs were not applied to Acute Effect benchmarks under the assumption that these higher-degree effects would be elicited even by chemicals that are not persistent. Chemicals with an aquatic half-life shorter than eight weeks were not considered to be persistent whereas those with a half-life of eight weeks or longer were considered persistent [15]. The aquatic persistence of each chemical was estimated using the BIOWIN 3 Ultimate Survey Model within Estimation Programs Interface (EPI) Suite [16]. By inputting the CAS number for each detected compound and loading the physical properties, BIOWIN 3 was used to gather a raw persistence output value. Then, using the BIOWIN 3 persistence value, biodegradation half-lives were estimated as described in Aronson et al. [17]. Chemical compounds with half-lives of less than eight weeks earned an ${AF}_{persistence}$ of 1 whereas chemicals with half-lives of 8 weeks or greater received an ${AF}_{persistence}$ of 5 [15].

In summary, using these three considerations (endpoint code, species requirement, and chemical persistence) for AFs, an overall AF was computed by multiplying ${AF}_{endpoint}\times{AF}_{species}\times{AF}_{persistence}$. Overall AF values ranged from 10 to 200. For each chemical the ECOTOX endpoint with the minimum concentration for each Benchmark type was divided by the overall AF calculated for the benchmark (Equation 3). For example, the overall AF for caffeine’s minimum benchmark was 20 which included the product of 10 for its AF_Endpoint_, 2 for its AF_Species_, and 1 for its AF_Persistence_. Due to variability in the type and number of ECOTOX screening values available for each chemical, the minimum ECOTOX benchmark was used to calculate the maximum TQ value for each chemical detection (equation 4). The most conservative ECOTOX benchmarks were evaluated to ensure the relevance and accuracy of the ECOTOX endpoints from which they were derived. In some cases, the minimum benchmark endpoints were misrepresentative; as a result, they were adjusted or removed. These adjustments are recorded in S10 Table.

$ECOTOX Benchmark= \frac{Endpoint concentration \left( \mu g/L \right)}{\left( \mathrm{AF}_{\mathrm{Endpoint}}\times\mathrm{AF}_{\mathrm{Species}}\times\mathrm{AF}_{\mathrm{Persistence}} \right)}$ (Equation 1)

$TQ= \frac{Measured concentration in sample ({\mu g}/L)}{ECOTOX-derived benchmark for chemical ({\mu g}/L)}$ (Equation 2)

# Disclaimer

Any use of trade, firm, or product names is for descriptive purposes only and does not imply endorsement by the U.S. Government.

# References

1. U.S. Geological Survey. Chapter A4. Collection of water samples [Internet]. 2006 [cited 2021 Mar 29]. Available from: https://doi.org/10.3133/twri09A4

2. U.S. Geological Survey. Chapter A3. Cleaning of equipment for water sampling [Internet]. 2004 [cited 2021 Mar 29]. Available from: http://pubs.er.usgs.gov/publication/twri09A3

3. U.S. Geological Survey. Chapter A5. Processing of water samples [Internet]. 2002 [cited 2021 Mar 29]. Available from: https://pubs.er.usgs.gov/publication/twri09A5

4. U.S. Geological Survey. The StreamStats program [Internet]. 2016 [cited 2021 Feb 17]. Available from: http://streamstats.usgs.gov

5. U.S. Environmental Protection Agency, U.S. Geological Survey. National Hydrography Dataset Plus - NHDPlus (Edition 2.10) [Internet]. Washington, D.C.: U.S. Environmental Protection Agency; 2012. Available from: http://www.horizon-systems.com/nhdplus/NHDPlusV2_home.php

6. U.S. Department of Agriculture-Natural Resources Conservation Service, U. S. Geological Survey, U.S Environmental Protection Agency. The Watershed Boundary Dataset (WBD) [Internet]. Fort Worth, Texas: U.S. Department of Agriculture, Natural Resources Conservation Service, National Cartography and Geospatial Center; 2009 [cited 2012 Jan 25]. Available from: http://datagateway.nrcs.usda.gov

7. Dewitz J. National Land Cover Database (NLCD) 2019 Products [Internet]. U.S. Geological Survey; 2021 [cited 2022 Jan 11]. Available from: https://doi.org/10.5066/P9KZCM54

8. D19 Committee. Practice for Performing Detection and Quantitation Estimation and Data Assessment Utilizing DQCALC Software, based on ASTM Practices D6091 and D6512 of Committee D19 on Water [Internet]. ASTM International; [cited 2021 Oct 7]. Available from: http://www.astm.org/cgi-bin/resolver.cgi?D7510-10R16E1

9. DeCicco LA, Corsi SR, Villeneuve DL, Blackwell BR, Ankley GT. toxEval: Evaluation of measured concentration data using the ToxCast high-throughput screening database or a user-defined set of concentration benchmarks. R Package version 1.0.0 [Internet]. 2018. Available from: https://code.usgs.gov/water/toxEval, doi:10.5066/P906UQ5I

10. U.S. Environmental Protection Agency. ToxCast & Tox21 Summary Files from invitrodb_v3.2 [Internet]. US EPA. 2017 [cited 2020 May 5]. Available from: https://www.epa.gov/chemical-research/exploring-toxcast-data-downloadable-data

11. Blackwell BR, Ankley GT, Corsi SR, DeCicco LA, Houck KA, Judson RS, et al. An “EAR” on Environmental Surveillance and Monitoring: A Case Study on the Use of Exposure–Activity Ratios (EARs) to Prioritize Sites, Chemicals, and Bioactivities of Concern in Great Lakes Waters. Environ Sci Technol. 2017 Aug;51(15):8713–24.

12. Corsi SR, De Cicco LA, Villeneuve DL, Blackwell BR, Fay KA, Ankley GT, et al. Prioritizing chemicals of ecological concern in Great Lakes tributaries using high-throughput screening data and adverse outcome pathways. Sci Total Environ. 2019 Oct 10;686:995–1009.

13. Olker JH, Elonen CM, Pilli A, Anderson A, Kinziger B, Erickson S, et al. The ECOTOXicology Knowledgebase: A Curated Database of Ecologically Relevant Toxicity Tests to Support Environmental Research and Risk Assessment. Environ Toxicol Chem. 2022 Jun;41(6):1520–39.

14. Pronschinske MA, Corsi SR, DeCicco LA, Furlong ET, Ankley GT, Blackwell BR, et al. Prioritizing Pharmaceutical Contaminants in Great Lakes Tributaries Using Risk-Based Screening Techniques. Environ Toxicol Chem. 2022;41(9):2221–39.

15. Hull RN, Kleywegt S, Schroeder J. Risk-based screening of selected contaminants in the Great Lakes Basin. J Gt Lakes Res. 2015 Mar 1;41(1):238–45.

16. U.S. Environmental Protection Agency. Estimation Programs Interface Suite [Internet]. Washington, DC; United States; 2012. Available from: https://www.epa.gov/tsca-screening-tools/epi-suitetm-estimation-program-interface

17. Aronson D, Boethling R, Howard P, Stiteler W. Estimating biodegradation half-lives for use in chemical screening. Chemosphere. 2006 Jun;63(11):1953–60.
