# Supplementary figures and images for: Evaluating pharmaceuticals and other organic contaminants in the Lac du Flambeau Chain of Lakes using risk-based screening techniques
Source: PLoS One. 2023 Jun 2;18(6):e0286571. doi: 10.1371/journal.pone.0286571 (PMC10237466; doi:10.1371/journal.pone.0286571)

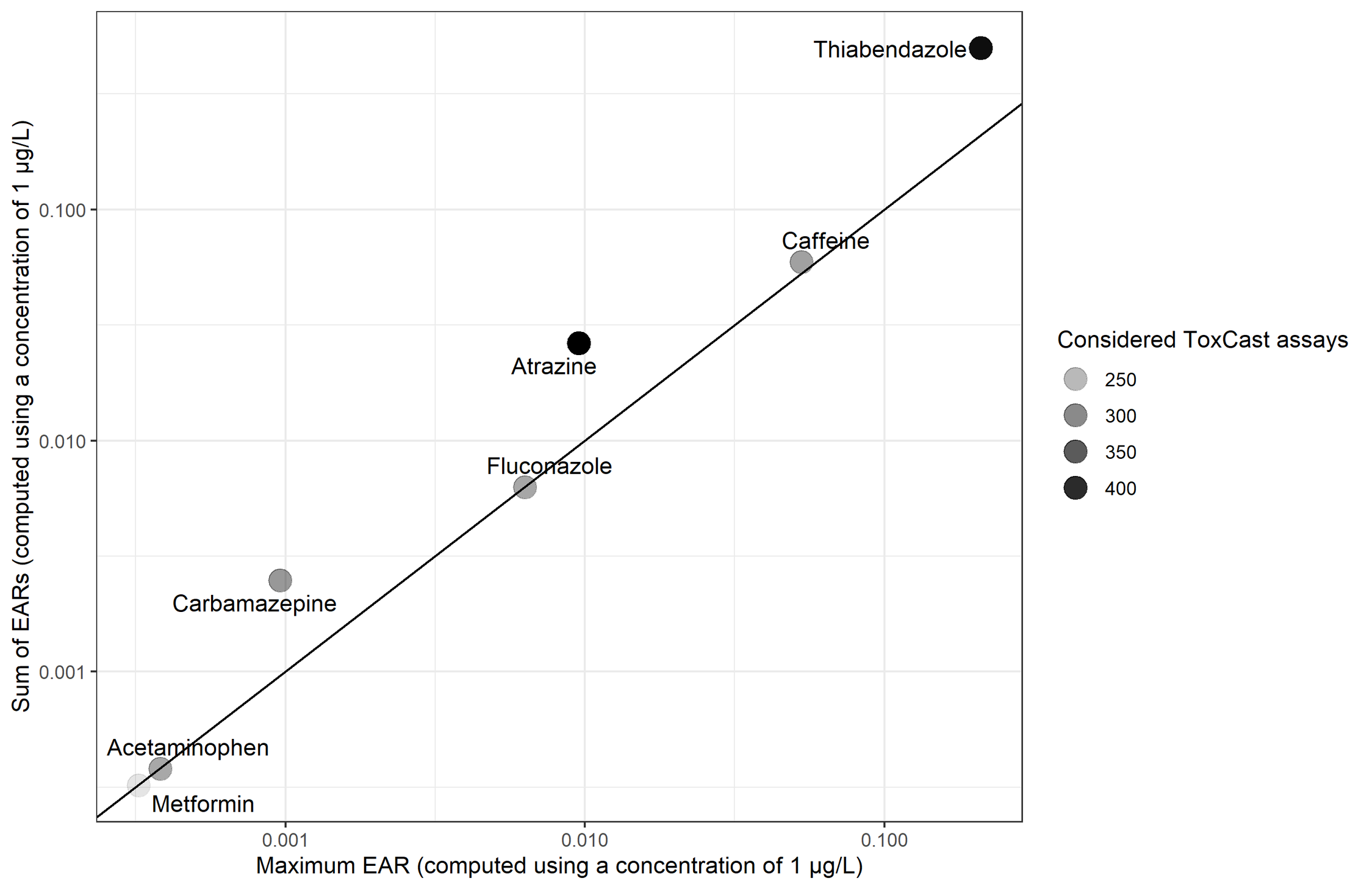

Supplement: S1 Fig — Comparison of the maximum Exposure-Activity Ratio (EAR) values (computed from minimum Activity Concentration at Cutoff values) and the sum of EARs (computed from all relevant Activity Concentration at Cutoff values) for chemicals detected in samples from the Lac du Flambeau Chain of Lakes, Wisconsin, USA, August 2020 –May 2021. The shade of each point is part of a gradient representing the number of assays considered for computing the EARs of each chemical. For the purposes of this comparison, EARs were computed using a constant, nominal concentration of 1 μg/L for all chemicals. (TIF) [file pone.0286571.s002.tif]

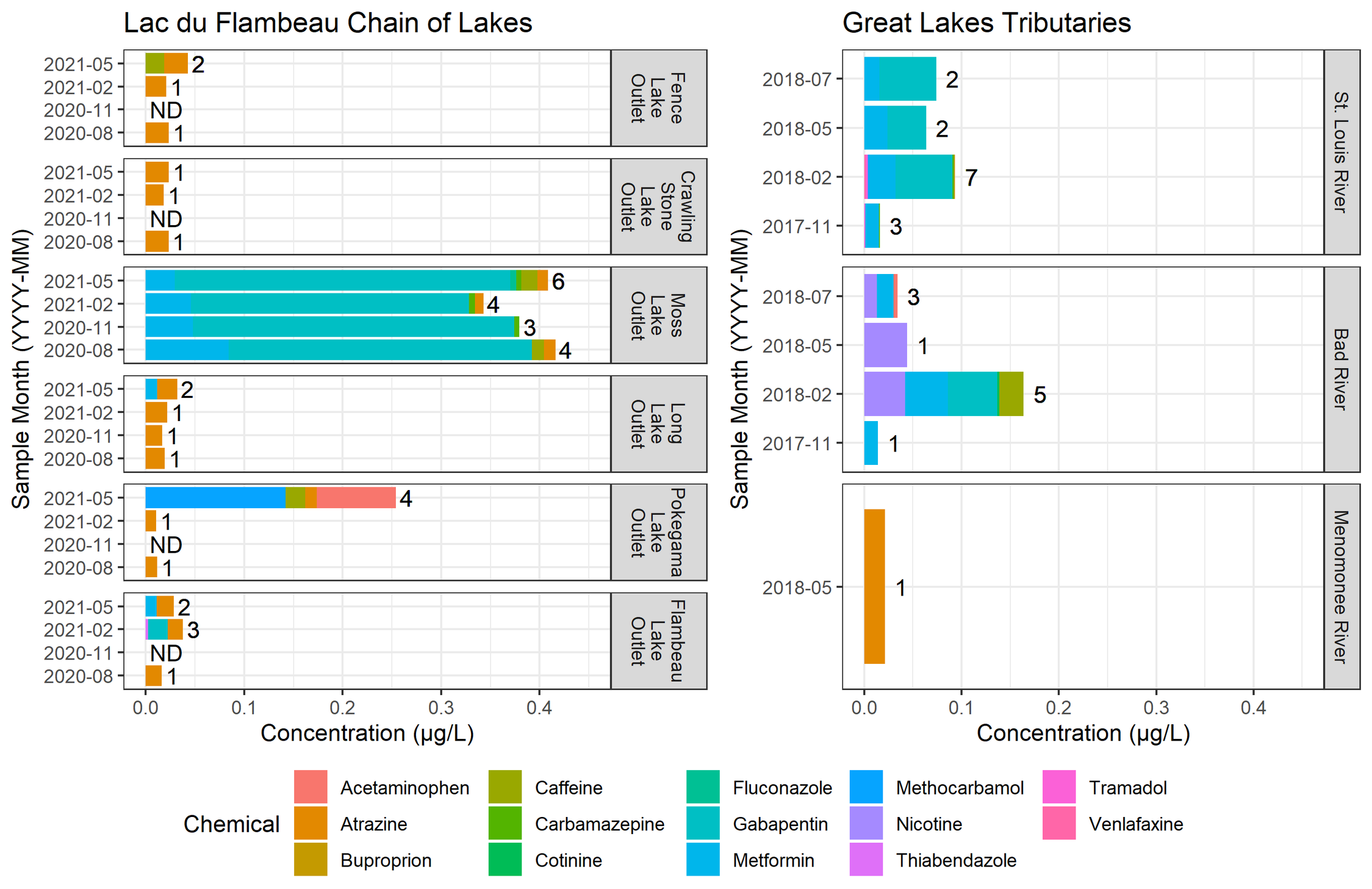

Supplement: S2 Fig — Surface water sample concentrations collected from the Lac du Flambeau Chain of Lakes, Wisconsin, USA, August 2020 –May 2021 (left). Surface water sample concentrations collected from three Great Lakes Tributary sites (USGS station IDs from top to bottom: 04024000, 04027000, 04087014), November 2017 –May 2018 (right). Sample concentration data are accessible from the National Water Information System. The three selected Great Lakes tributary sites capture drainage areas with similar wastewater contributions and land cover profiles. Gabapentin concentration values are all considered to be estimates. All samples were analyzed for using the same analytical procedures at the USGS National Water Quality Laboratory. The number of unique chemicals detected in each sample is indicated to the right of each bar. “ND” was used as a label for samples which lacked chemical detections. (TIF) [file pone.0286571.s003.tif]
